# The Interplay Between the Genetic and Immune Landscapes of AML: Mechanisms and Implications for Risk Stratification and Therapy

**DOI:** 10.3389/fonc.2019.01162

**Published:** 2019-11-07

**Authors:** Lourdes M. Mendez, Ryan R. Posey, Pier Paolo Pandolfi

**Affiliations:** ^1^Department of Medicine and Pathology, Cancer Research Institute, Beth Israel Deaconess Cancer Center, Beth Israel Deaconess Medical Center, Harvard Medical School, Boston, MA, United States; ^2^Ludwig Center at Harvard, Harvard Medical School, Boston, MA, United States

**Keywords:** acute myeloid leukemia, genetics, immune landscape, metabolism, targeted (selective) treatment, immunotherapy

## Abstract

AML holds a unique place in the history of immunotherapy by virtue of being among the first malignancies in which durable remissions were achieved with “adoptive immunotherapy,” now known as allogeneic stem cell transplantation. The successful deployment of unselected adoptive cell therapy established AML as a disease responsive to immunomodulation. Classification systems for AML have been refined and expanded over the years in an effort to capture the variability of this heterogeneous disease and risk-stratify patients. Current systems increasingly incorporate information about cytogenetic alterations and genetic mutations. The advent of next generation sequencing technology has enabled the comprehensive identification of recurrent genetic mutations, many with predictive power. Recurrent genetic mutations found in AML have been intensely studied from a cell intrinsic perspective leading to the genesis of multiple, recently approved targeted therapies including IDH1/2-mutant inhibitors and FLT3-ITD/-TKD inhibitors. However, there is a paucity of data on the effects of these targeted agents on the leukemia microenvironment, including the immune system. Recently, the phenomenal success of checkpoint inhibitors and CAR-T cells has re-ignited interest in understanding the mechanisms leading to immune dysregulation and suppression in leukemia, with the objective of harnessing the power of the immune system via novel immunotherapeutics. A paradigm has emerged that places crosstalk with the immune system at the crux of any effective therapy. Ongoing research will reveal how AML genetics inform the composition of the immune microenvironment paving the way for personalized immunotherapy.

## Introduction

Acute myeloid leukemia (AML) is a disease of the myeloid lineage in which rapidly proliferating progenitors are transformed, leading to a block in terminal differentiation and the ability to self-renew, the latter normally being the privilege of only hematopoietic stem cells. Two decades of research have demonstrated that these expanded clones are the result of a differentiation hierarchy which mirrors normal hematopoiesis, with “stem-like” progenitors giving rise to partially committed myeloid progenitors, themselves giving rise to committed but undifferentiated myeloid precursors (1). This dysfunctional hierarchy, referred to as malignant hematopoiesis, occurs at the expense of normal hematopoiesis and is characterized by progressive cytopenias and immunosuppression with concomitant increased risk of infection and bleeding.

AML is an aggressive disease; in the U.S. from 2009 to 2015, the 5-years survival rate was 28.3% (2). For decades the backbone of therapy for fit patients has been intensive induction chemotherapy, such as the 7 + 3 regimen with the combination of an anthracycline and infusional cytarabine. Despite complete remission rates of 60–80% in younger patients and 40–60% in older patients with such regimens (3–5), rates of overall survival are far more sobering highlighting the critical problem of relapsed leukemia, following an initial response. Thus far, the only exception to this somber picture is acute promyelocytic leukemia (APL), which as a result of the high rates of cure achieved with ATRA- and ATO- containing therapy, is considered separately from non-APL AML for the purpose of risk classification/stratification (6). For patients with intermediate or high risk disease who are fit, allogeneic stem cell transplantation offers the chance of durable remission and long term survival weighed against treatment related morbidity and mortality (7, 8). Elderly patients and/or patients with multiple comorbidities, have faced more limited options mainly hypomethylating agents (HMA) and low dose cytarabine (6) until recently. The last few years have seen a flurry of FDA approvals for AML including a liposomal formulation of daunarubicin and cytarabine, the anti-CD33 antibody drug conjugate gemtuzumab ozogamicin, the IDH1/2 mutant inhibitors ivosidenib and enasidenib, the FLT3-ITD inhibitors midostaurin (in combination with chemotherapy) and gilternitib and the BCL2 inhibitor venetoclax (in combination with HMA or low dose cytarabine).

Efforts over the last years to understand the impact of recurrent genetic alterations in AML on prognosis have resulted in genetic classification systems that summarize the risk of relapse following intensive induction chemotherapy (3, 6). A similar system is not available to guide treatment with HMAs. Identifying the predictors, genetic and others, of response and resistance to the recently approved targeted agents, such as the IDH1/2 mutant inhibitors and the BCL2 inhibitor, venetoclax, is an active field of research. Aberrant mitochondrial metabolism and the regulation of mitochondrial apoptosis have emerged as central themes in the discussion about response and resistance to existing and emerging therapies; how these processes tie into the genetics of AML remains largely unknown. Durable remissions following allogeneic transplantation for the treatment of leukemia was an early success story in the history of immunotherapy, demonstrating that leukemia could be eradicated by an effective immune response. The successes of checkpoint inhibitors in solid tumor oncology has resulted in a renaissance for immunotherapies against AML with promising modalities including cellular therapies, such as dendritic cell fusion vaccines to address minimal residual disease (9, 10). However, there is a considerable gap between the overwhelming progress in basic research in AML and advances in AML immunotherapy. While the genetics of AML have been extensively studied to classify and predict responses to chemotherapy, the relationship between genetic aberrations and the immune system has yet to be comprehensively described. This review aims to cover this topic, as well as how existing therapies affect the immune milieu. While the immune modulatory properties of a number of genetic mutations present in AML would seem to be an obvious connection, this remains an emerging area of investigation in AML. The experience to date suggests that co-occurring mutations together with additional key modifiers, such as stage of differentiation and metabolic profile, will converge on the immune microenvironment dictating biology and prognosis.

## Genetics of AML and Risk Stratification

It has been appreciated for some time that outcomes with intensive induction chemotherapy are quite variable, and that a minority of patients attain durable remissions. Older age (>60 years old) and certain genetic alterations predict for markedly inferior outcomes. Multiple studies have demonstrated the prognostic utility of classifying patients by genetic mutations and cytogenetic abnormalities (11–14). The ability of the ELN genetic classification system, to discriminate outcomes, was reported by Mrozek et al. in 2012 in 1,550 adult patients treated with intensive chemotherapy. The overall survival at 3 years varied in younger patients (<60 years), from 66% in the favorable risk group to 10% in the high risk group compared to the older patients (>60 years), 33% in the favorable risk group and 3% in the high risk group. Such variability in outcomes has been understood to reflect underlying heterogeneity in disease biology and propelled efforts to further define this heterogeneity (11). The advent of next-generation sequencing technology and ensuing insight into the genetic and clonal architecture of AML, opened a new chapter in AML pathogenesis, classification and risk stratification.

Large-scale sequencing efforts have provided a catalog of recurrently mutated genes in AML. Whole exome and whole genome sequencing performed on 200 *de novo* AML samples by The Cancer Genome Atlas Network revealed that AML is characterized by few mutations in coding genes, on average 13 per patient, 5 of these recurrent mutations (15). Recurrently mutated genes can be grouped into functional categories revealing mutual exclusivity between different combinations of mutations. This suggests that alterations of different genes may converge on common pathways to give rise to AML. Mutual exclusivity was observed between, but is not limited to, mutations in *NPM1, CEBPA, RUNX1, TP53*, and transcription factor-fusion genes. Co-occurrence of mutations was also noted, such as among *FLT3-ITD, DNMT3A*, and *NPM1* (15). Interestingly, RNAseq expression data revealed clustering that correlated with FAB subtypes and thus stage of differentiation, in accordance with other publications (15–17).

A large data set of 1,540 patients, treated on one of three German-Austrian AML Study Group trials, integrated clinical data with genetic profiling (cytogenetics and sequencing of 111 driver mutations) enabling a more detailed view of the mutational landscape of AML, prompting a new proposed genomic classification scheme for diagnosis beyond the current WHO subgroups, and allowing the authors to tackle the problem of the prognostic implications of co-occurring mutations. Analysis of allele frequencies allowed for establishment of clonal relationships identifying mutations in the epigenetic modifiers *ASXL1, TET2, IDH1/2*, and *DNMT3A* as the earliest event occurring in the founding clone whereas mutations in receptor tyrosine kinase-RAS pathway genes occurred late as previously described (18–20) with more than one such mutation in a given patient (12). The proposed, new classification system is composed of 11 genomic subgroups of AML including AML with *NPM1* mutation; AML with mutated chromatin, RNA-splicing genes or both; AML with *TP53* mutations, chromosomal aneuploidy or both; AML with inv (16) or *t* (16, 16); AML with biallelic CEBPA mutations; AML with *t* (8, 21); AML with *MLL* fusion genes, AML with inv (3); *t*(3, 3), GATA2, MECOM; AML with *IDH2-R172* and no other class-defining lesions; AML with *t* (6, 9). The chromatin-spliceosome and TP53-aneuploidy groups in particular represent new genomic subgroups with their respective class defining lesions imparting a deleterious effect on survival. Interestingly, the initial, recently reported findings of the Beat AML programme found that *TP53* and *ASXL1* (one of the recurrent mutated chromatin genes) were associated with a general pattern of drug resistance in an *ex vivo* 122 small molecule inhibitor screen (21).

The results of efforts over the last years to understand the impact of recurrent genetic alterations on outcomes following intensive chemotherapy are summarized in the updated ELN 2017 genetic classification system for AML (6). The ELN 2017 system starts to incorporate knowledge about the impact of co-occurring mutations on outcome; specifically, the favorable prognosis of *NPM1*-mutated AML is noted to be contingent on a low *FLT3-ITD* mutational burden. At the present time, the ELN 2017 system does not include other interactions between/among genes in its risk stratification algorithm and this remains a frontier in AML that is actively being explored (12, 21). The complexity of mutation co-occurrence is such that the negative prognostic impact of FLT3-ITD may be most relevant to AML with the most frequent three-gene co-occurrence of mutations in *NPM1, DNMT3A*, and *FLT-3* whereas, the negative impact on survival in AML with *FLT3-ITD* and *NPM1* mutation or *FLT3-ITD* and *DNMT3A* mutation is less pronounced irrespective of the FLT3-ITD allelic frequency (12).

More recently, single cell sequencing-based assays have been performed to finely resolve clonal and subclonal architectures in AML, offering insights into clonal evolution during both leukemogenesis and disease progression following treatment (22, 23). Van Galen et al. recently applied single cell RNAseq and genotyping to profile 40 bone marrow aspirates (from 16 AML samples, five healthy controls). By transcriptomics analysis, they identified six malignant cell types that resembled normal bone marrow cell types and correlated with leukemia cell differentiation state: HSC-like, progenitor-like, GMP-like, promonocyte-like, monocyte-like, conventional dendritic cells-like. The composition of a patient's leukemia with respect to the malignant cell types varied considerably and could be predominantly composed of one cell type or contain the spectrum of cell types. The abundance of malignant cell types correlated with morphologic and cell surface marker metrics in clinical use. Moreover, gene signatures for each malignant cell type allowed interrogation of the composition of the TCGA AML sample collection revealing close correlations with the genetics of AML. Intriguingly, they further found that monocyte-like AML cells express various immunomodulatory genes and inhibit T cell activation *in vitro*, suggesting that this malignant cell type participates in the immunosuppressive leukemia microenvironment (23). This study represents a pioneering effort to capture and integrate multiple facets of AML biology—differentiation state, intratumoral heterogeneity, genetics and the immune milieu in AML. In spite of a growing wealth of genetic data, at the present time, comparatively little is published on the immunologic consequences of AML genetic perturbations, representing a significant gap in our knowledge of AML biology.

## Features of Immune Dysregulation in AML

AML was among the first malignancies successfully treated with “adoptive immunotherapy,” a term coined by G. Mathe in 1965 referring to allogeneic stem cell transplantation (24). This provided proof-of-concept that leukemia could be eradicated by an effective immune response, the graft-vs.-leukemia effect, and also implicated a suppressed endogenous anti-leukemia immune response as part of the pathogenesis of AML. The successes of immune check point inhibitors in solid tumors has invigorated research into immunotherapies, that like allogeneic stem cell transplantation, could result in durable remissions, with the hope that novel, selected adoptive cell therapies and biologics would additionally be better tolerated (9, 25).

Components of a successful anti-tumor immune response include engagement of the innate immune system via danger signals, presentation of tumor-associated antigens to T cell receptors (TCR) together with activating co-stimulatory signals and immunologic memory (26). In AML, remodeling of the leukemia microenvironment and barriers to an effective immune response include but are not limited to (1) low neoantigen burden and defective antigen presentation, (2) imbalance between T effector (Teff) and regulatory populations (Treg) in favor of regulatory/suppressive cells, (3) T cell exhaustion, such as through upregulation of immune checkpoint ligands and receptors, chronic inflammation, (3) increased myeloid derived suppressor cell populations (MDSC), (4) increased suppressive macrophage populations, and (5) production of immunosuppressive soluble factors including metabolites (9, 25). Multiple agents including biologics, selected adoptive cell therapies and targeted agents are currently the subject of clinical investigation in an effort to address the ineffective anti-leukemia immune response. A non-exhaustive list is provided as an example, along with the proposed immunologic effect in Table S1; a subset of these agents is briefly discussed below.

Eliciting specific antitumor immunity requires the presentation of polypeptide fragments of tumor-associated antigens bound to major histocompatibility complex (MHC) class I or II, in conjunction with co-stimulatory signals to a cognate CD8^+^ (MHC I bound antigen) or CD4^+^ (MHC II bound antigen) TCR. MHC class I is expressed by most cells and presents self-antigens, whereas MHC class II expression is normally restricted to professional antigen presenting cells, such as dendritic cells, B cells and macrophages and binds extracellular antigens. The critical role of antigen presentation in AML is highlighted by a recent publication reporting that relapse following allogeneic stem cell presentation is associated with down-regulation of MHC class II genes (27). Neoantigens are thought to be the ideal tumor antigens in part because the corresponding TCR repertoire may have escaped negative selection during central tolerance. As discussed later in this review, neoantigens encoded by recurrent AML mutations have been identified, including those derived from NPM1c, FLT3-ITD, and mutant p53. High mutational/neoantigen burden correlates with response to checkpoint blockade (28, 29). With an average of 13 mutations/patient (15), AML is considered to be a malignancy with low mutational burden and thus, at least in this sense, low immunogenicity. On the other hand, genetic subsets, such as *TP53*-mutated AML or AML with loss of *TP53* may be associated with an increased mutational burden and complex karytotype and thus, increased epitopes, which could predict for a higher likelihood of response to checkpoint blockade (30). In addition, the efficacy of allogeneic stem cell transplantation in a subset of patients indicates, that at least within certain contexts, AML is sufficiently immunogenic.

In addition to the challenges imposed by low mutational/neoantigen burden, multiples studies have reported on defective antigen presentation by AML cells and APC in the leukemia microenvironment (25). AML cells are aberrant myeloid precursors with their normal counterparts maturing into dendritic cells, monocytes, macrophages and neutrophils. Dendritic cells and macrophages are professional APC while monocytes and neutrophils though not professional APC, have been described to participate in antigen presentation and stimulation of the adaptive immune response (31–35). As discussed in the next section, multiple reports substantiate the ability of HMA to enhance antigen presentation pathways in AML and recalibrate the immune microenvironment to elicit anti-tumor immunity (Table S1). AML vaccines are an emerging, promising modality of immunotherapy that may address the problem of antigenicity/immunogenicity (Table S1). Vaccine strategies include neoantigen vaccines (36), leukemia-derived dendritic cell vaccines (37), GM-CSF transduced/secreting leukemia cell vaccines (38) and modified, autologous dendritic cell-based vaccines. AML-dendritic cell fusion vaccines have the benefit of overcoming defective antigen presenting functions of leukemic cells and of not requiring a priori selection of antigens. Moreover, by presenting multiple antigens with the potential to engage multiple TCRs, a diverse T cell response could be elicited (39, 40).

AML has been demonstrated to induce suppressive populations derived from the myeloid lineage, such as myeloid derived suppressor cells (MDSC) and M2 macrophages. MDSC are associated with a poor prognosis in solid and hematologic malignancies and have been associated with a blunted response to immunotherapy (41, 42). These immature myeloid cells suppress Teff through arginase-1, nitric oxygen synthase and ROS production (43). Pyzer et al. demonstrated that MDSC are increased in the peripheral blood of patients with AML compared to normal controls. Interestingly, MDSC appeared to be derived from both normal progenitors and leukemia cells in line with previous reports that AML cells employ arginase to suppress Teff (44–46). Given that HMA are known to possess differentiation activity, an open question is whether differentiation of blasts into leukemia-derived dendritic cells, macrophages or neutrophils down-regulates MDSC function originating from blasts and renders leukemia more immune competent. If this is indeed the case, it would have implications for approved and investigational targeted agents that induce differentiation of blasts, such as the recently approved selective inhibitors of IDH1/2-mutants.

The nomenclature M1/M2 macrophages, refers to the distinction between “classically activated” macrophages, M1, that engage in effective anti-microbial or tumor responses and “alternatively activated” macrophages, M2, that have anti-inflammatory activity and tumor supporting properties. AML promotes the polarization of leukemia-supporting macrophages (47). Yang and colleagues further found that, in patients, leukemia associated macrophages are heterogeneous, with both M1- and M2-like leukemia associated macrophages (48). M2-like leukemia associated macrophages were associated with a poor prognosis. In murine models of leukemia, treatment with polyIC (a synthetic analog of double stranded RNA) or inhibition of the SAPK/JNK pathway “repolarized” leukemia associated macrophages toward an M1 phenotype and resulted in prolonged survival.

Regulatory T cells (Treg) constitute another part of the immune suppressive leukemia microenvironment. Treg restrain immune competent helper T and cytotoxic T cells through inhibition of T cell proliferation and cytokine production and play an important role in preventing autoreactivity (49, 50). Treg also restrain the activity of B cells (51) and cells of the innate immune system (52, 53). Increased Treg compared to normal controls are present in AML patients at diagnosis, during chemotherapy-induced pancytopenia and following chemotherapy; the pretreatment Treg level has been observed to correlate with response to chemotherapy. Interestingly, the lowest levels of Treg were observed at the time of hematopoietic recovery following chemotherapy (54–57). Importantly, the available data indicates that interventions directed against Treg render chemotherapy and immunotherapy more effective in hematologic malignancies (49, 58). One strategy employed to address the suppressive leukemia microenvironment in which regulatory or suppressive cell types dominate, is to tip the scale through infusion of either manipulated autologous or allogeneic cells with cytolytic activity, such as chimeric antigen receptor T cells (CAR-T cells; see Table S1) (59).

**Figure 1:**
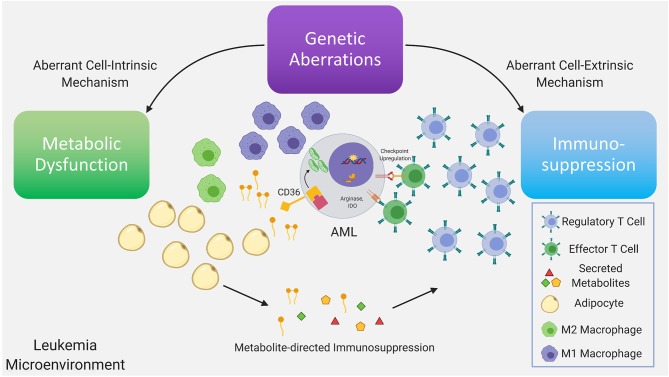
Genetic aberrations drive dysfunctional cell-intrinsic and -extrinsic signaling, with the former having direct metabolic consequences and the latter having direct immunologic consequences. Further dysregulation is driven via interactions between the altered metabolome and immune system as a consequence of the existing immunometabolic network. Recurrent genetic mutations found in AML have been demonstrated to promote T regulatory cell expansion, suppress proliferation of T effector cells, skew macrophage maturation toward the suppressive M2 phenotype. A subset of these immune suppressive mechanisms are mediated by metabolites. In AML, depletion of amino acids, such as tryptophan and asparagine via increased arginase-1 and IDO levels in leukemia cells, M2-macrophages and MDSC, limits T effector activity. Furthermore, mobilization of fatty acids in the leukemia microenvironment from adipocytes, a subset of which can function as pro-inflammatory lipid mediators, may simultaneously provide anti-apoptotic and immunosuppressive signals inhibiting T effector activity and promoting suppressive immune cell types. In leukemia cells the metabolic regulatory network that is stimulated by adipocytes may include upregulation of the lipid scavenger receptor CD36, the fatty acid activated receptor/transcription factor PPARG, the fatty acid binding protein (FABP4) along with BCL2. Upstream genetic determinants/mutations regulating specific metabolic adaptations in leukemia cells is the subject of ongoing research.

Another means by which T cell effector function is restrained is T cell exhaustion, which was initially characterized in the context of chronic viral infection, but is by now a well-established mechanism cancer immune evasions (60). Cell surface expression of inhibitory co-receptors marks exhausted T cells. In contrast to the co-stimulatory signal required for naïve T cell activation in concert with TCR MHC-peptide engagement, signaling through inhibitory ligands/receptors, results in decreased T cell proliferation and cytokine production (Figure 1). The best studied inhibitory receptors are CTLA-4 and PD-1; others include Tim-3 and TIGIT (9). Inhibitory ligands/receptors are upregulated during acute infection/inflammation to limit the inflammatory response. Type I and II interferons induce the expression of PD-L1/2, as part of non-neoplastic immune circuits (61–63); interferons have also been reported to induce the expression of PD-L1/2 on AML blasts (64–66). Immune checkpoint inhibitors (ICI) that prevent signaling through inhibitory ligands and receptors, have been approved for the treatment of various solid tumors (67). Increased expression of inhibitory receptors/ligands including CTLA-4, PD-1, PD-L1/2 on T cells and myeloid cells has been described following treatment with HMA in patients with myeloid neoplasms (68, 69) and forms part of the rationale for ongoing clinical trials investigating HMA combined with ICI. It may be necessary to disable multiple inhibitory pathways, as reported by a preclinical study in which inhibition of CTLA-4, PD-1, and LAG-3 was required for adoptive T cells to exert an anti-leukemia effect (70).

As noted above, soluble factors, such as arginase 2 have been implicated in the induction of a tolerogenic environment by AML blasts and M2 macrophages (9). Arginase 2 is the mitochondrial version of the enzyme and functions in wound repair together with nitric oxide synthase (NOS), with the two enzymes having the same substrate, L-arginine (71). Indoleamine 2,3 dioxygenase (IDO), that causes the breakdown of tryptophan, is another enzyme that has been linked to an immunosuppressive leukemia microenvironment. IDO may be involved in the increased Treg population and function observed in AML (9). Fatty acids and lipid mediators derived from fatty acids, are another class of metabolites that bridge cellular metabolism with immune signaling (72) (Figure 1). Their role in modulating the balance between an active and suppressed immune milieu in leukemia is currently under investigation (73). For example, the lipid mediator PGE2, has been demonstrated to promote tumor progression through induction and maintenance of MDSC (74, 75) with PGE2 inducing PD-L1 expression on tumor associated macrophages and MDSC (76). Strategies to target lipid mediator signaling include inhibition of PGE2 receptors and could potentially involve pharmacologic modulation of the PPAR nuclear receptor family whose natural ligands include lipid mediators (73). Targeting lipid mediators may represent a novel therapeutic approach for various malignancies (77) including AML.

## Immunomodulation and Anti-leukemia Therapy

Alongside the recognition that immune evasion is a hallmark of cancer (78) has emerged a paradigm in which tumor clearance by antineoplastic therapy by de facto must mobilize an effective anti-tumor immune response. Conventional therapies, like chemotherapy, have been revisited with renewed interest in their capacity to elicit immunogenic cell death and anti-tumor immunity (58). There is a growing literature on the mechanisms by which DNA demethylating/hypomethylating agents (HMA) reprogram leukemia cells and refashion the leukemia immune microenvironment. Likewise, there is increasing appreciation that targeted agents address cell-extrinsic mechanisms promoting an immune suppressive leukemia microenvironment. Below are highlights regarding the immune modulatory dimension of chemotherapy, HMAs and targeted agents. Notably, despite the general paucity of data on the genetic determinants of the leukemia immune microenvironment and response to immunotherapy, in the case of these three categories of therapy, there are clear links between therapy and AML genetics potentially suggesting the specific immunologic space in between.

A small subset of chemotherapeutic agents and fractionated radiation therapy can induce “immunogenic cell death” (79, 80). Immunogenic cell death (ICD) refers to apoptosis, that results in coordinated activation of the immune system and antigen-specific immunity (81), as compared to the “silent” cell death that occurs at steady state as part of the physiologic turnover of normal cells. Activation of the ER stress pathway/Unfolded Protein Response and ensuing emission of danger signals are defining characteristics of ICD. Damage associated molecular patterns (DAMPs) include surface–exposed ER chaperones (calreticulin, HSP70 and HSP90). DAMPs are recognized by pattern recognition receptors (PRRs) present on macrophages, monocytes and dendritic cells, leading to activation of the innate arm of the immune system. These chaperones, when exposed to the cell surface, can have antigenic peptides bound that are endocytosed by PRRs, leading to cross presentation of antigens to T cells (82–84). Notably, a number of anthracyclines, some of these a mainstay of AML therapy, have been demonstrated to induce ICD (85–90). Interestingly, type I interferon signaling downstream of toll-like receptor 3 (TLR3) signaling, is required for anthracycline-induced ICD in a murine breast cancer model.

Expression of DAMPs, PRRs has been explored as predictive biomarkers to ICD-inducing chemotherapy. In a prospective cohort of 50 patients with AML, Fucikova et al. addressed the effect of anthracycline-based chemotherapy on the surface expression of various DAMPs and their relation to leukemia-specific T cell immunity (91). They demonstrated that AML blasts, but not normal CD45^+^ CD33^+^ cells from healthy volunteers displayed exposed calreticulin, HSP70 and HSP90 independent of exposure to induction anthracycline-based chemotherapy suggesting a baseline level of ER-stress response in a subset of AML; in line with this, surface exposed calreticulin correlated with the expression of genes involved in ER stress response. Noting that surface calreticulin is best known for promoting phagocytosis by antigen-presenting cells and thus initiating anti-tumor immunity, the authors showed that upon recovery from chemotherapy, the normal PBMCs from patients expressing high surface calreticulin, as compared to low surface calreticulin, showed a gene expression pattern reflecting enrichment of TH1 polarization, T cell activation, CD8^+^ T-cell cytotoxicity and NK-related genes. Consistent with the gene expression data, surface calreticulin^hi^ patients had increased circulating NK cells at the time of recovery from chemotherapy. Additionally, they had an increased fraction of CD8^+^ and CD4^+^ T cells responding to leukemia associated antigens both prior to and following chemotherapy as well as an increased percentage of T central memory cells, again both prior to and at the time of recovery from chemotherapy (91).

A cornerstone of therapy for patients unable to tolerate intensive induction chemotherapy has been DNA-demethylating agents, also called hypomethylating agents (HMA) i.e., azacitadine and decitabine. Both drugs are cytidine analogs and were originally developed as cytotoxic agents before being found, at low doses, to cause DNA demethylation by inhibition of DNA methyltransferase-1 (DNMT1) (92). DNA demethylation has been demonstrated in cell lines to cause reactivation of genes, leading to differentiation (93) and this is one of the proposed mechanisms of therapeutic activity in myeloid neoplasms including AML. However, DNA methylation/methylcytosine has not been found to correlate with response in patients with AML (94, 95). In addition to its effect on DNA, azacitadine has been reported to inhibit the RNA methyltransferase, DNMT2 (96) suggesting that it's mechanism of action in myeloid neoplasms may be linked to modification of RNA. Consistent with this possibility, in a recent report, distinct RNA 5-methylcytosine methyltransferase chromatin complexes were demonstrated to mediate sensitivity vs. resistance to azacitadine (97). Up-regulation of antigen presentation pathways, such as MHC expression, are part of the mechanism by which HMA are described to recalibrate the immune micoenvironment promoting anti-tumor responses (98–103).

Clinically, the optimal response to HMA has been noted to require multiple cycles of treatment in line with a cell-intrinsic mechanism involving slow kinetics, potentially related to the differentiation of leukemic cells (104) and akin to other differentiation therapies (105). Differentiation is associated with cell cycle arrest and apoptosis, but one could speculate that an additional benefit could be enhanced presentation of antigens, as is seen with the maturation of professional antigen presenting cells and/or diminished MDSC-like activity thus leading to more effective engagement of the immune system. Decitabine has also been demonstrated to alter Treg maturation and activate the expression of endogenous retroviruses resulting in engagement of the dsRNA response/interferon-beta pathway thus, suggesting a non-cell autonomous mechanism of action is also at play in AML (101, 102, 106–108). Indeed, preclinical data indicates that hypomethylating agents potentiate the response to anti-CTLA-4 immunotherapy (101) and a dendritic cell/AML fusion vaccine (109) with ongoing clinical trials in AML currently exploring the combination of HMA and checkpoint inhibitors (NCT02890329) or dendritic cell/AML fusion vaccine.

The combination of all-trans retinoic acid (ATRA) and arsenic trioxide (ATO) for the treatment of APL is widely considered to constitute the first targeted therapy for cancer as well as the first example of differentiation therapy. ATRA targets the RARA moiety and ATO the PML moiety of the PML-RARA fusion protein for degradation in *PML-RARA* APL (110). The phenomenal success of this chemotherapy-free targeted combination therapy (111) was informed by genetic models providing insights into the underpinnings of resistance and response to ATRA and ATO (112). The road to this success is at once instructive and a reminder of the promise of targeted therapies. Remarkably, ATRA has well-documented immune modulatory activity with effects on CD4 and CD8 T cell development and dendritic cells (113–119) including in preclinical murine models of APL, as a single agent and in combination with DNA vaccines (120, 121). ATRA's potential as a chemical adjuvant, has been explored in various studies (118, 120, 121). In a cohort of patients with metastatic renal cell carcinoma treated with 7 days of ATRA followed by interleukin 2, ATRA was noted to reduce the number of immature myeloid suppressor cells by day 7, approaching the levels of these cells seen in normal controls; this effect or lack thereof was noted to correlate with plasma concentration of ATRA. In this study, ATRA also improved the ability of peripheral blood mononuclear cells to stimulate allogeneic T cells (122). As regards ATO, this drug has been shown to selectively deplete Treg *in vitro* and *in vivo* by inducing the expression of pro-oxidative genes in Treg with concomitant induction of increased levels of nitric oxide and reactive oxygen species (ROS). ATO-induced Treg depletion appeared to be dependent on “oxidative and nitrosative bursts.” Consistent with these observations, the authors found that in a model of colon cancer, a reduction in tumor volume was observed in immunocompetent but not athymic NUDE mice (123). Xu et al. examined the effect of ATO treatment on Treg from APL patients and likewise found that the number and function of Treg was attenuated by ATO treatment (124). ATO has also been reported to up-regulate NK ligands on tumor cells (125) modulate NK cell receptors toward enhanced NK cell mediated cytolytic activity (126). Taken together, the data on the immune modulatory activities of ATRA and ATO raise the possibility that these targeted agents could be employed in the treatment of subsets of AML, beyond APL, because of their immune modulatory activity. In this regard, ATRA and ATO could be considered together or in novel combinations as recently suggested by preclinical studies in *IDH1/2*-mutated AML (127, 128). As discussed in the next section, targeted agents against recurrent genetic mutations in non-APL AML, likewise display significant affects against components of the suppressive leukemia immune microenvironment.

It has become apparent that all categories of anti-neoplastic drugs can modulate the immune response. Approved targeted therapies possess highly specific immune modulatory activities pertinent to their potential roles in the induction, consolidation and maintenance phases of therapy. Ongoing delineation of the immunologic mechanisms of action of targeted therapies is expected to inform the rationale combination of targeted therapies, as well as, novel combinations with chemotherapy, HMA, cellular therapies and biologics.

## Genetic Determinants of Immunologic Remodeling

Recurrent genetic mutations found in AML have been intensely studied from a cell intrinsic perspective. By comparison the literature on cell extrinsic mechanisms of action of these mutations is still building and is heavily based on studies of solid tumors. Nonetheless, the mechanisms by which recurrent genetic mutations in AML, such as *NPM1, FLT3-ITD, IDH1/2*, and *TP53* can modulate the immune microenvironment in AML, are steadily being elucidated (Figure 1).

### NPM1c

*NPM1* is among the three most frequently mutated genes in AML, along with *FLT3-ITD* and *DNMT3A*, occurring in 30–35% of *de novo* AML (6, 15). The NPM1 protein shuttles between the nucleus and cytoplasm (129) and is most prominently located in the nucleolus (130). More recently, in an elegant series of experiments Nachmani et al. demonstrated that NPM1 regulates 2′-O-methylation of rRNA through snoRNA binding which is required for normal hematopoietic stem cell maintenance; *Npm1*-deletion in hematopoietic stem cells leads to a bone marrow failure syndrome and proclivity to develop leukemia (131). In 2005 Falini et al. discovered *NPM1*-mutated AML reporting that in 35.2% of 591 bone marrow specimens of patients with de novo AML, NPM1 was located in the cytoplasm as a result of a mutation in the cytoplasmic portion of the protein (NPM1c) (132). NPM1c AML was noted to be associated with a normal karyotype and response to induction chemotherapy. FLT3-ITD co-occurs with NPM1 mutation in ~40% of cases (132, 133). FLT3-ITD (VAF of >50%) negates the positive prognosis associated with NPM1c (38, 134–137). NPM1-mutated AML in the absence of FLT3-ITD in CR1 does not derive RFS or OS benefit from allogeneic stem cell transplantation (7, 136) underscoring the chemosensitivity and likely immunogenicity (as discussed below), of this subtype of AML. Notably, in the Phase 1b dose escalation and expansion study of venetoclax combined with HMA in elderly patients with AML, *NPM1*-mutated AML was associated with a remarkably high CR + CRi [91% (138)]. This result remains to be confirmed in larger studies, but is suggestive a decreased threshold for apoptosis. Given multiple publications indicating that NPM1c elicits endogenous T cell immunity, another pending question is whether the high rates of CR and the duration of response following venetoclax combined with HMA, is linked to tumor-specific immunity. Finally, the persistence of *NPM1*-mutated transcripts has been demonstrated to be a powerful biomarker for disease relapse (139).

The favorable prognosis of NPM1-mutated AML in the absence of FLT3-ITD has been posited to be at least partially mediated by engagement of the immune system (140). In particular, the unique C-terminal sequences of mutated NPM1 in *NPM1*-mutant AML have been investigated as potential leukemia specific antigens that may elicit endogenous T cell responses and could also serve as targets for immunotherapy. Noting that aberrant localization of mutant NPM1 in the cytoplasm could lead to processing by the class I degradation pathway and subsequent presentation by HLA, Liso et al. tested and confirmed that mutated-NPM1 peptides can be presented by HLA molecules (141). Greiner et al. demonstrated that mutant NPM1 is immunogenic with T cells from the peripheral blood of patients with *NPM1*-mutant AML reacting *in vitro* to specific peptides derived from the mutant NPM1 (142). Notably, in a patient with molecular relapse of *NPM1-mutant* AML, donor lymphocyte infusion displayed mutant-NPM1 specific T cell responses that were linked to molecular remission (140). Subsequently, Forghieri and colleagues investigated NPM1-mutated specific T cell response in *NPM1-mutant* AML patients over time. They observed that a robust response could be identified following attainment of CR post-induction chemotherapy and was sustained in patients with a durable remission compared to a low number or absence of NPM1-mutated specific T cells in patients with leukemia relapse suggesting that a mutated NPM1-specific T cell response is inversely correlated with MRD and relapse (143). Van der Lee et al. characterized the HLA 1 ligandome of primary AMLs to investigate whether mutant NPM1 is a neoantigen in AML. Multiple peptides were identified from mutant NPM1 in the precipitated ligandome. Mutant NPM1 is characterized by a 4 bp hotspot insertion in exon 12 that results in a mutant protein that is 4 amino acids (aa) longer than the wildtype protein with the c-terminal 11 aa, CLAVEEVSLRK, translated in an alternative reading frame. 5 mutant NPM1-derived peptides were identified in the HLA 1 ligandome in *NPM1*-mutant but not *NPM1-wildtype* AML. The CLAVEEVSL peptide, predicted to bind to HLA-A^*^02:01, was chosen for further characterization. As tumor-specific T cells have previously been described in healthy individuals, the authors utilized PBMCs from healthy HLA-A^*^02:01–positive individuals to isolate CD8+ T cell specific for CLAVEEVSL and then further identified clones that were reactive against HLA-A^*^02:01–positive primary AMLs with mutant NPM1. Finally, the authors tested the potential of CLAVEEVSL as a target for TCR gene transfer and found that T cells transduced with the TCR for mutant NPM1 resulted in killing of *NPM1-mutant* AML cells *in vitro* and *in vivo*, notably, with a survival benefit observed in the latter experiments (144).

### FLT3-ITD

FMS-like Tyrosine Kinase 3 (FLT-3) is a receptor tyrosine kinase, normally involved in hematopoietic stem and progenitor cell proliferation and survival (145). It is mutated in ~30% of AML cases and is known to confer adverse prognosis (146). Mutations in *FLT3* are observed as either point mutations in the tyrosine kinase domain (TKD) or as an internal tandem duplication of the juxtamembrane domain (ITD), with the latter more frequently occurring than the former (147). It is known that the direct consequence of *FLT3-ITD* mutations is strong, constitutive activation of signaling, and that this activation is insensitive to negative feedback via SOCS1 (148). It is appreciated that while FLT3-ITD utilizes downstream pathways active during normal FLT3 signaling, downstream signaling becomes deregulated, as observed with the aberrant activation of STAT5 signaling (149). In addition to being less frequent, TKD mutations confer a less striking phenotype clinically, being less strongly associated with poor prognosis and are even associated with a favorable prognosis in combination with NPM1 mutations (150).

Constitutive activation of FLT3 via internal tandem duplication (ITD) mutation or point mutations in the tyrosine kinase domain (TKD) has been shown to mediate cell-extrinsic changes in the immune landscape of AML. Mice with a total body ITD mutation demonstrate expanded populations of dendritic cells (DCs), dendritic cell precursors (pre-DCs), and regulatory T cells (Treg) as a result of the ITD mutation (151). The expansion of dendritic cells and dendritic cell precursors was demonstrated to be intrinsic to ITD-mutant hematopoietic cells in experiments utilizing bone marrow transplant (BMT) chimera mice. The subsequent expansion of Treg observed in total body ITD-mutant mice was corroborated in BMT chimeras and shown to be a cell-extrinsic effect given that the T cells were of Flt3 wild-type origin. Interestingly, effector T cell (Teff) populations were expanded as well, although the ratio of Treg to Teff within the total T cell population was higher as compared to wild-type chimeras. Scurfy mice, which possess a truncated, non-functional variant of FOXP3, rapidly develop a lethal autoimmune condition due to insufficient immunoregulation caused by a deficiency of Tregs. Immunologically relevant genotypes crossed with these mice either exasperate or ameliorate this condition, resulting in increased or decreased survival of these mice. In their study, Lau et al. crossed ITD and scurfy mice, and observed significantly more rapid lethality, due to accelerated autoimmunity. This strongly suggests that the cell-extrinsically expanded Teff population is able to perform effector functions in the absence of the enriched Treg population.

These results mirror similar observations in patients with AML, where expanded populations of dendritic cells and dendritic cell precursors containing the ITD mutation have been observed (152). A prospective study tracking these populations in patients at first diagnosis and in remission following chemotherapy demonstrated that the populations of expanded dendritic cell precursors persist after patients enter remission (153). The group noted that persistence of pre-DCs was associated with increased risk of relapse in patients with the ITD mutation. Midostaurin, a tyrosine kinase inhibitor that potently targets both wildtype and mutated FLT3 has demonstrated immune-modulatory effects with implications on patient prognosis following remission (154). A reduction in CD4^+^, CD25^+^ cells, and in the mRNA level of FOXP3, consistent with a decrease in Treg, was observed following treatment in AML patients treated with Midostaurin, a possible consequence of ITD inhibition (155).

Since *FLT3* mutations are associated with increased risk of relapse following treatment with chemotherapy, this patient subset is often treated with allogeneic hematopoietic stem cell transplant (HSCT) in first remission (156). Several groups have noted that the reduced risk of relapse following HSCT is directly linked to the graft-vs.-leukemia (GVL) effect (157, 158). A recent study demonstrated production of IL-15 by leukemia cells in patients with *FLT3* mutations treated with Sorafenib, a tyrosine kinsase inhibitor which targets mutant FLT3, similar to Midostaurin (159, 160). The production of IL-15 was specific to patients with the ITD mutation, as non-ITD AML cells or ITD cells not treated with Sorafenib did not express the same level of IL-15. The effect was abrogated by depletion of CD8+ T cells but not NK cells, indicating that the increased alloreactivity is due to changes in the Teff population. Antibody-based depletion of IL-15 or transfer of *Il15ra*-deficient T cells to recipient mice blunted the GVL effect and subsequently resulted in failure to control disease. Mechanistically, the increase in IL-15 was due to decreased ATF4 activity, resulting in an increase in IRF7 phosphorylation. Knockdown of IRF7 or overexpression of ATF4 resulted in abrogated IL-15 production. Competitive Kinobead pulldown of Sorafenib binding targets in patient samples implicated FLT3 as a possible mediator of ATF4 expression. Accordingly, the authors proposed a mechanism by which FLT3 activity in ITD-mutated patients suppresses IL-15 production in AML blasts via ATF4 inhibition of IRF7. These results were corroborated in patients treated with Sorafenib, as responders achieving complete remission demonstrated increased levels of IL-15, p-IRF7, and IFN-g following treatment as compared to non-responders, for whom remission was not attained, who did not mirror this effect.

### IDH1/2-mutations

Mutations in Isocitrate Dehydrogenase 1 and 2 (*IDH*1 and *IDH2*, respectively) account for between 9 and 20% of mutations in AML (15). These mutations result in production of the oncometabolite R-2-Hydroxyglutarate (R-2-HG) from alpha-Ketoglutarate (a-KG) (161). In the context of AML, R-2-HG accumulation is known to be responsible for a blockade in terminal differentiation (162), epigenetic rewiring of AML blasts (163, 164), and increasing genome instability resulting in acquisition of subsequent mutations (165). Recently, patients with relapsed, refractory *IDH1*- and *IDH2*-mutant AML were demonstrated to have significant ORR and CR rates to the novel, selective IDH1- and IDH2-mutant inhibitors, ivosidenib and enasidenib, with evidence of differentiation of blasts, in a subset of patients leading to a differentiation syndrome akin to the previously described differentiation syndrome described in patients with APL treated with ATRA and ATO, in accordance with the predicted mechanisms of actions of these novel inhibitors (105, 166). Interestingly, preclinical models also indicate that *IDH1/2*- mutant AML also demonstrate sensitivity to, ATRA and ATO, the targeted combination therapy employed in APL (128).

The production of the oncometabolite R-2-Hydroxyglutarate (R-2-HG) mediates the proto-oncogenic effects of mutant IDH1 and IDH2. Human T cells express multiple transporters capable of importing R-2HG, including SLC13A3 and SLC22A6 (167). The impact of R-2-HG on the immune response has been characterized largely in gliomas and it remains to be seen whether there are cancer-specific mechanisms of R-2-HG immune suppression. In gliomas, levels of R-2-HG present in the extracellular space can be as great as 5-fold higher than within tumor cells and can be transported intracellularly to T cells via these receptors (168). Extracellular R-2-HG is taken in rapidly regardless of activation status of T cells. This enantiomer demonstrated dose-dependent inhibition of T lymphocyte proliferation, while S-2HG had no such effect (166). This inhibition of T cell proliferation was rescued by addition of the SLC13A3 inhibitor NAA. Mechanistically, R-2-HG caused reduced calcium influx and subsequently suppressed NFAT translocation and proliferation. R-2-HG treated T lymphocytes were also shown to have reduced mitochondrial fitness and ATP production. A syngeneic glioma model of wild-type and *IDH1*-mutated cancers demonstrated no change in survival in *RAG2* knockout mice, indicating that suppression of anti-tumor response mediates the competitive advantage of R-2-HG production *in vivo*. Interestingly, T lymphocyte polarization was shown to be affected in the context of AML as compared to gliomas, with exogenous addition of R-2-HG interfering with HIF-1a stability and skewing the development of Th17 cells toward Treg cells (169). Subsequent work has demonstrated that IDH1-mutant gliomas correlated with lower expression of the T cell attracting chemokines CXCL9 and CXCL10, and subsequent reduction in number of CD3^+^, CD8^+^ tumor infiltrating lymphocytes (166, 170, 171). Both total and phosphorylated STAT1 protein was shown to be reduced in IDH1 mutant gliomas, and knockdown of STAT1 in wildtype *IDH1* tumors resulted in reduced expression of these chemokines, suggesting that R-2-HG can potentially abrogate cytokine signaling through this pathway. The fraction of Foxp3^+^/CD4^+^ cells within gliomas was not observed to change with IDH status (166). Interestingly, the R172K mutation in *IDH2* has the unique functional ability to impact T cell development, increasing the amount of double negative (CD4^−^ CD8^−^) and decreasing the fraction of double positive thymocytes, unlike that of other mutations in *IDH1* and *IDH2*, as a result of the exceptionally high levels of intracellular R-2-HG this mutation produces (172).

R-2-HG has also been shown to modulate the bone marrow microenvironment and the innate immune system. In addition to reduced T lymphocyte chemotaxis, mutant IDH gliomas also exhibit reduced influx of neutrophils in murine models, as well as a reduced expression of innate immune chemotactic molecules including CCL2, CCL3, CXCL1, CXCL2, CXCL4, CXCL16, GM-CSF, IL1RA, IL-2, IL-6 (166). In the microenvironment of gliomas, R-2HG is able to suppress activation of the C5 component of the complement system and subsequent complement-mediated phagocytosis, although it does not directly affect dendritic cell differentiation from bone marrow mononuclear cells and subsequent antigen-presenting function to T lymphocytes. However, reduced levels of CD83 were observed on human monocyte-derived dendritic cells in addition to differential metabolic changes and decreased IL-12 secretion in response to LPS stimulation. Natural Killer cell ligands and subsequent tumor cell lysis has also been shown to be reduced in *IDH1*-mutated gliomas (173). Within the bone marrow microenvironment, R-2-HG is taken up by stromal cells, where it inhibits TETs as well as induces activation of the transcription factors NF-kB and GATA (174).

### TP53-mutation

The p53 tumor suppressor is a transcription factor that regulates multiple, critical cellular functions including cell division, DNA damage and repair, apoptosis, senescence, metabolism (175–179). It is one of the most commonly mutated genes in cancer (180). Though best known for its role in regulating the response to DNA damage and apoptosis thus functioning as a guardian of the genome, studies indicate that p53 may function as a tumor suppressor in a non-cell autonomous fashion via modulation of the immune system and inflammation.

AML with *TP53* mutations are present in ~6% of patients with AML and can be combined into a genomic category with chromosomal aneuploidy (13% of AML) as the two types of genetic alterations are closely correlated (12). The incidence of *TP53* mutation in AML with complex karyotype (3 or more chromosomal abnormalities) has been reported as 69–78% (181). Missense point mutations and small deletions occur (15) in addition to allelic loss through deletion of the short arm of chromosome 17 (182). *TP53* mutated AML, particularly in combination with adverse cytogenetics, has a dismal prognosis following chemotherapy (30, 183). Among driver mutations, mutations in *TP53* are second only to inv (3), *GATA2* and *MECOM(EVI1)* mutations in terms of the increased risk of death associated with the mutation (12) likely reflecting, at least in part, the chemoresistance associated with *TP53* mutations (184). *TP53* mutations are also associated with decreased survival and an increased risk of relapse following allogeneic stem cell transplantation (185–187) with *TP53* mutations detected at relapse indicating that the *TP53* mutated clone was involved in relapse (187). The poor outcomes associated with *TP53* mutated AML following allogeneic stem cell transplantation could be mediated by the persistence of minimal residual disease and/or resistance to graft vs. leukemia effect and thus negative modulation of the anti-leukemia immune response.

Patients with *TP53* mutated AML tend to be older (188) and as such, more likely to be intolerant of intensive induction chemotherapy, making them more likely to be treated with hypomethylating agents. Interestingly, in a single institution study of patients with AML treated with the hypomethylating agent, decitabine, that sought to understand whether specific somatic mutations predicted response to therapy, *TP53* mutation was highly correlated with bone marrow blast clearance albeit with incomplete mutation clearance. In this study, a *post-hoc* analysis indicated that overall survival was not negatively affected by unfavorable risk cytogenetic abnormalities nor by the presence of *TP53* mutations and *TP53* status did not affect outcome following transplantation (95). In patients ineligible for intensive cytotoxic therapy and/or allogeneic stem cell transplantation, the addition of venetoclax to hypomethylating agents represents one of the most exciting and promising novel combination therapies. In the phase 1b dose-escalation and expansion study of the BCL2 inhibitor, venetoclax, combined with azacitadine or decitabine, in elderly patients with AML, the rate of CR + CRi, the median duration of CR + CRi and the median overall survival in patients with *TP53* mutation compared to all comers was 47%, 5.6, 7.2 months vs. 67%, 11.3 and 17.5 months (138).

P53 mediates the cellular response to multiple stressors including DNA damage, oncogene activation and viral infection and there is evidence of interplay among these three insults mediated by p53. It has been suggested that in addition to guarding genomic integrity, tumor suppressors, such as p53, also guard the integrity of tumor immunosurveillance (189, 190). Many viruses, including oncoviruses, have developed mechanisms to directly or indirectly disrupt p53 function underscoring the critical role of p53 in antiviral immunity and the suppression of virus-associated cancers. In line with this, in a mouse model bearing an extra copy of *TP53*, enhanced resistance to viral infection was observed (191–193). P53 activates transcription of critical regulators of the innate immune response, including genes involved in pathogen sensing, such as Toll-like receptors 3 and 8 (194) and type I IFN. Notably, upregulation of Toll-like receptors is induced by p53 in response to DNA-damaging agents in human primary cells and cell lines (192) and differential regulation of TLRs is observed between wildtype and mutants of p53 which retain transactivation capacity (193). The immunostimulatory effects of radiation therapy and certain chemotherapeutic agents has been proposed to be mediated via p53- activated TLR and IFN type 1 pathway (195) and by extension, dysregulation of these pathways downstream of mutated p53 may mediate resistance to these therapies in multiple malignancies including *TP53*-mutated AML. P53 can also activate natural killer cell ligands, ULBP1 and 2 on cancer cells leading to enhanced recognition and killing by NK cells (196, 197).

P53 senescence programs have been implicated in the resolution of inflammation (198) with chronic inflammation being a well-established risk factor for inflammation-associated epithelial malignancies. A non-cell autonomous mechanism of tumor suppression by p53 has been described through skewing of macrophage polarization toward the tumor-inhibiting M1-subtype; selective p53 deletion in the myeloid lineage led to elevated levels of inflammatory cytokines and a significant increase in tumor initiation whereas mild activation of p53 in the myeloid lineage restricted tumor progression (199, 200). It remains to be seen whether p53 inactivation or mutation in AML recapitulates the non-cell autonomous mechanisms observed in epithelial tumor models leading to increased inflammatory cytokines and M2-macrophages associated with disease initiation and progression or whether impaired regulation of TLR signaling and interferon pathways is implicated in the initiation or maintenance of TP53-mutated AML. Another element of p53 function is regulation of metabolism with promotion of OXPHOS (201). Interestingly mutations in mitochondrial-encoded electron transport genes, is associated with mutated p53 in patients with AML (202). Additional studies are needed to clarify whether mutated p53 is a driver of aberrant mitochondrial metabolism in this subset of AML contributing to remodeling of the leukemia microenvironment and chemoresistance. p53 reactivating therapies are currently in clinical trials in AML and may shed light on the role of p53 in the anti-leukemia immune response.

P53 and PML share multiple overlapping functions, such as the response to various stressors (oxidative, DNA damage, viral infection), regulation of senescence and apoptosis, regulation of fatty acid oxidation (203–205). Moreover, the two proteins have been shown to functionally and physically interact (206). PML is a key cofactor for p53 transcriptional function in part through recruitment of p53 into PML nuclear bodies (NB) where p53 undergoes post-translational modification (207–209). In APL, where PML-RARA disrupts normal NB formation and function, one would predict that p53 functions, including immunoregulatory activities, would be compromised. Indeed, it has been demonstrated that engagement of a PML-p53 checkpoint is key to the efficacy of ATRA and ATO in APL, downstream of NB re-formation (210). Hence, APL serves as an example of p53 dysfunction in the wildtype p53 setting, which has been well-documented in non-APL AML (211, 212) and raises the question of whether immune dysregulation secondary to inactivation of p53 pathways plays also a role in the pathogenesis of APL, and in its profound response to therapy upon the restoration of the p53-PML crosstalk.

### *JAK2V617F* and Others

While the above mutations have been studied most extensively with respect to their immunomodulatory properties, a modicum of data exists for a handful of additional, recurrently altered genes in AML. For example, the *JAK2V617F* mutation, most commonly associated with the Myeloproliferative Neoplasm (MPN) Polycythemia Vera (PV) which can progress to AML, has been shown to regulate expression of PD-L1 cell-intrinsically (213). The authors demonstrated that this was dependent on constitutively active JAK2/STAT3/STAT5 signaling as a result of the *V617F* mutation. Secondary AML evolving from an MPN has a dismal prognosis with a median overall survival of 2.5–7 months (214). The JAK1/2 inhibitor, Ruxolitinib, is currently in clinical trials in combination with Decitabine (NCT02257138) or chemotherapy (NCT03878199) in an effort to improve outcomes for this subset of AML patients. The results of the phase II study of Ruxolitinib plus Decitabine reported an ORR of 61%, CRi 11%, and 17% of patients (3/18) proceeding to transplant (215). Ruxolitinib is also currently under investigation in combination with venetoclax for the treatment of relapsed, refractory AML (NCT03874052). *Jak2V617F* was shown to cooperate with loss of Dnmt3a, one of the top three mutated genes in AML, to induce myelofibrosis (MF) and inflammatory signaling in bone marrow HSPCs (216). Similarly, mutated *RUNX1*, estimated to occur in ~13% of human AML cases, has been demonstrated to modulate NF-kb signaling in a cell intrinsic manner, and has been proposed to be able to promote inflammatory signaling in the bone marrow microenvironment (217). Finally, in metastatic melanoma (MM), mutations in *NRAS* are associated with poor response to immune checkpoint blockade, suggesting yet another mechanism of immune dysfunction that could result from this mutation in AML (218, 219).

The genetic determinants of the leukemia immune microenvironment are starting to be elucidated. Forthcoming reports may yield insight as to whether favorable risk AML subgroups, currently defined in genetic terms, are enriched for a favorable immune profile as complement, as one might imagine for *NPM1*-mutated AML with wildtype *FLT3*. By comparison, the current high-risk genetic subgroups of AML may have in common the enrichment of a suppressive immunologic milieu supported by non-cell autonomous mechanisms as has been suggested for *FLT3-ITD*.

## Oncometabolism, Inflammation, and Response to Therapy

The altered lipid composition of AML cells was first reported in the 1970s (220). Recent publications further substantiated an altered lipidome in the plasma and bone marrow of patients with AML (221, 222). Lipids function as signaling molecules for a variety of immune-related processes. Multiple studies support a dual role for fatty acids and fatty acid derived lipid mediators in sustaining malignant cells and modulating antitumor immune responses toward tolerance (73).

Among the reported metabolic changes in AML are increased mitochondrial mass and oxygen consumption, but decreased spare reserve relative to normal hematopoietic cells (223) and thus, increased vulnerability to metabolic perturbations. Within the mitochondria, the Krebs cycle and fatty acid oxidation (FAO) generate metabolites that feed into the electron transport chain, donating electrons to form a gradient which powers the production of ATP, with oxygen acting as the electron acceptor through a process called, oxidative phosphorylation (OXPHOS). Fatty acids are obtained by cells from the extracellular environment or catabolized from intracellular sources. Within the mitochondria, during FAO, fatty acids undergo iterative cycles of catabolism producing FADH2 and NADH and acetyl CoA during each cycle; FADH2 and NADH feed directly into the electron transport chain and thus OXPHOS (224). In solid tumors, FAO has been reported to support energy requirements during times of stress (224, 225) and promote chemoresistance (226). In AML, FAO has been reported to modulate the threshold for apoptosis and regulate quiescence in leukemic progenitors (227). In a model of blast crisis CML, Ye and colleagues found that leukemia stem cells (LSC) enriched in visceral adipose tissue, display a pro-inflammatory phenotype that drives lipolysis. They further demonstrated that LSC in adipose tissue have high FAO with fatty acid uptake regulated by the scavenger receptor, CD36. CD36^+^ LSC were shown to be chemoresistant consistent with the findings by Farge et al. on increased FAO, up-regulated CD36 and an OXPHOS signature in pre-existing, residual AML cells that survive cytarabine treatment in a cohort of patient derived xenografts (228). LSC from patients with relapsed AML, but not de novo AML, employ FAO as a means of sustaining OXPHOS to avoid cell death induced by combination therapy with HMA and the BCL2 inhibitor, venetoclax (229). Transcriptomics analysis of chemoresistant leukemia cells reveals an immune/inflammatory stress response gene signature highlighting the dynamic between mitochondrial metabolism/fatty acids and the immune component of the microenvironment (228).

**Figure 2:**
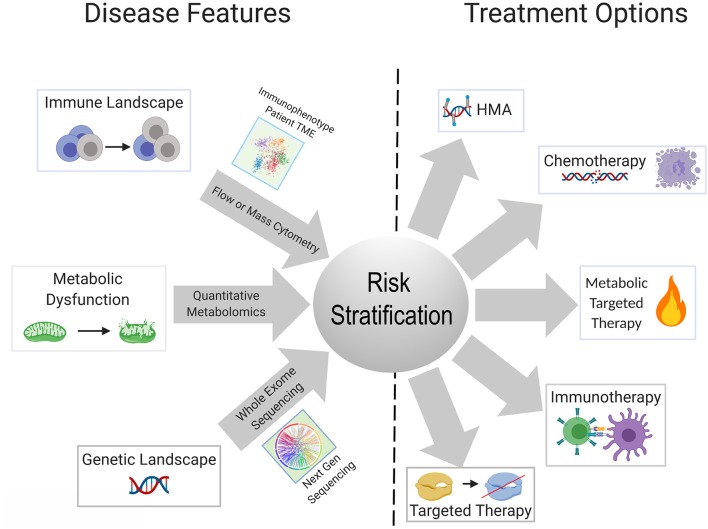
Schematic showing possible integration of available tools for improved risk stratification to inform therapeutic strategy for an individual patient. Advances in next gen sequencing technology, metabolite quantification methods, and widespread use of cytometry-based immunophenotyping, will continue to improve the feasibility of these techniques as diagnostic tools. Better understanding of the relationships and dependencies between the immunologic, metabolic, and genetic perturbations seen in AML can allow for refined usage of available and emerging therapeutics and combinations thereof.

Metabolic reprogramming in cancer cells is achieved by genetic mutations and altered signaling (230). One gap that is starting to be addressed in this field is which genetic mutations central to the pathogenesis of AML are able to reprogram the lipidome. Stuani et al. reported that in a cell line model of *IDH1*^*R*132*H*^ AML, proteomic, lipidomic and isotope-labeled glucose and glutamine approaches to profile the metabolic status of *IDH1*-mutated vs. wildtype cells, revealed that mutant IDH1 directs high lipid anabolism (231). Similarly, the *FLT3-ITD* mutation is associated with specific metabolic perturbations including promotion of the Warburg effect (232, 233). Mutations in mitochondrial-encoded electron transport genes, are associated with mutated p53 (202). Interestingly, localization of leukemia to adipose tissue was observed by Ye et al. in the blast crisis *BCR-ABL* CML model, but not an *MLL-AF9* murine model, indicating that genetics and perhaps cell of origin may regulate the metabolic phenotype of leukemia (234). In normal hematopoietic stem cells, we previously reported that FAO is critical in cell fate determination and regulated by *Ppard* downstream of *Pml* (235). The Andreef group used a bone marrow-derived adipocyte and acute monocytic leukemia co-culture system, to interrogate the genetic and metabolic networks that are stimulated in monocytic leukemia by adipocytes under conditions of limiting nutrients. They found that adipocytes activate a transcriptional network that includes *PPARG* and its target genes *CD36, FABP4*, and *BCL2* (236). The PPAR family of nutrient-activated steroid hormone receptors thus, appears to represent one family of transcription factors regulating fatty acid metabolism that carries over from normal to malignant hematopoiesis. There remains a paucity of information on the genetic determinants of metabolic reprogramming and the apparent concomitant changes in inflammation/immune response. A substantial body of literature exists delineating the interaction between AML metabolism, leukemia cell survival, response to existing therapies and novel therapeutic strategies, extensively reviewed elsewhere (237, 238). Mounting evidence indicates that identifying the upstream regulators and genetic determinants of AML metabolism will have clear implications for understanding how the genetic and immune landscapes of AML are interconnected as well as inform risk stratification and therapeutic algorithms in patients with AML (Figure 2).

## Discussion

The genetic landscape of AML has come into focus through large scale sequencing efforts. Together with intensive study of cell-intrinsic mechanisms of leukemia initiation and maintenance driven by recurrent mutations, this has led to the development and approval of multiple targeted agents for the treatment of AML. Approximately 50 years after the observation that “adoptive immunotherapy,” that is allogeneic stem cell transplantation, is effective in the treatment of leukemia, a myriad of biologics and cellular therapies are under investigation for the treatment of AML. Following in the steps of solid tumor biology, we have learned that immune modulation is not limited to biologics and cellular therapy and rather is also seen with targeted agents consistent with a paradigm in which eliciting anti-tumor immunity is a cornerstone of effective antineoplastic therapy.

The explosive growth in the armamentarium of approved and investigational agents for the treatment of AML has outstripped our current classifications systems for AML. Though there are snapshots of cell-extrinsic mechanisms of immune remodeling induced by recurrent AML mutations, which we have described in this review, a panoramic view of the AML immune landscape awaits the results of ongoing large scale profiling efforts of AML patient samples. Collaborative efforts have the potential to integrate the genomic and immunologic landscapes of large numbers of patients with AML with clinical outcomes. Combined with transcriptomic, and perhaps metabolic data, this will undoubtedly lead to revised classification systems for the diagnosis, risk stratification and treatment of patients with AML (Figure 2). Diagnostic procedures at the time of initial diagnosis and in the event of relapse, will almost certainly evolve to include assessment of the immune milieu and potentially metabolic profiling, in addition to existing morphologic, immunophenotypic and cytogenetic/molecular genetic testing. Concomitant assessment of leukemia and the immune milieu is accessible via flow cytometry based immunophenotyping (239) while the application of technologies, such as mass cytometry has yet to be incorporated into clinical practice. Metabolic characterization of leukemia and its niche can be performed through metabolomics analysis employing NMR spectroscopy or mass spectrometry or through the measurement of single metabolites of interest (240). Evaluation of the metabolic profile of the leukemic microenvironment could identify actionable metabolic targets ultimately resulting in immunomodulation that complements cytotoxic, targeted or immunotherapy.

The crosstalk between recurrent genetic alterations in AML and the immune system and the net outcome following treatment may be significantly influenced by leukemia metabolism modulating the threshold for cell death and effecting changes in the leukemia microenvironment promoting leukemia survival. Successfully employing the spectrum of approved and emerging therapies in AML, in rational combinations and sequences toward personalized medicine, will require algorithms incorporating “immunologic” risk alongside genetic risk. Ongoing, mechanistic insights into the links between the genetics and immunologic status of subtypes of AML are expected to inform selection of therapy(s) that can converge on vulnerabilities, including metabolic, of a given subtype, resulting in elimination of leukemia.

## Author Contributions

All authors listed have made a substantial, direct and intellectual contribution to the work, and approved it for publication.

### Conflict of Interest

PP serves in the Scientific Advisory Board of Agios Pharmaceuticals. The remaining authors declare that the research was conducted in the absence of any commercial or financial relationships that could be construed as a potential conflict of interest.
