# Supplementary material for: The Interplay Between the Genetic and Immune Landscapes of AML: Mechanisms and Implications for Risk Stratification and Therapy
Source: Front Oncol. 2019 Nov 7;9:1162. doi: 10.3389/fonc.2019.01162 (PMC6856667; doi:10.3389/fonc.2019.01162)
Supplement: Supplementary file 1 [file Table_1.pdf]

Class	Therapeutic	Immune Effect
**Biologics**	Checkpoint inhibitors	Block inhibitory signal of T cell activation --> Reinvigorate T cell cytotoxic activity.
	Anti-CD38	Induce Antibody & Complement-dependent cellular cytotoxicity (ADCC) by immune cells
	IL2 (alone or as part of immunocytokine) +/- cellular therapy	Stimulate NK cells, macrophages, neutrophils and anti-tumor T cell response
	Peptide(s) vaccines +/- adjuvant or HMA	Elicit antigen-specific T-cell immunity
	anti-CXCR4 antibody	Induce Antibody & Complement-dependent cellular cytotoxicity by immune cells
**Cellular Adoptive Therapy**	Allogeneic Dendritic cells	Restore antigen presenting function of DC to innate and adaptive immune cells
	Autologous Dendritic cell vaccines	Restore antigen presenting function of DC to innate and adaptive immune cells
	Allogeneic Wilms Tumor (WT1) CTL	Antigen-specific cytolytic activity against WT1-expressing leukemia
	Allogeneic NK cells	Non-MHC recognition and tumor cell killing
	NKR-2 CAR T-cells	Recognition of stress ligands through NK cell receptor (NKR-2), direct killing and immunomodulation
	anti-CD123 or anti-CD33 CAR T-cells	Direct killing of antigen expressing cells
**Targeted Therapies**	DNMT inhibitors/Hypomethylating Agents	Enhanced immunogenicity of leukemia cells, decreased Treg
	All-trans retinoic acid (ATRA)	Dendritic cell maturation. Adjuvant promoting tumor-specific T cell immunity.
	Oxidative Phosphorylation Inhibitor	Immunomodulation of OXPHOS dependent immune cell subsets

Table 1: Approved and investigational immunomodulatory agents that are being studied alone or as part of combination therapy in clinical trials.
